# Are advance directives helpful for good end of life decision making: a cross sectional survey of health professionals

**DOI:** 10.1186/s12910-017-0197-6

**Published:** 2017-06-05

**Authors:** Eimantas Peicius, Aurelija Blazeviciene, Raimondas Kaminskas

**Affiliations:** 1 Department of Social Sciences and Humanities, Medical Academy Lithuanian University of Health Sciences, Mickevičiaus g. 9, Kaunas, LT-44307 Lithuania; 2 Department of Nursing and Care, Medical Academy Lithuanian University of Health Sciences, Mickevičiaus g. 9, Kaunas, LT-44307 Lithuania

**Keywords:** Advance directives, End of life, Medical decision making, Medical ethics, Nurses, Physicians, Pharmacists, Lithuania

## Abstract

**Background:**

This paper joins the debate over changes in the role of health professionals when applying advance directives to manage the decision-making process at the end of life care. Issues in relation to advance directives occur in clinical units in Lithuania; however, it remains one of the few countries in the European Union (EU) where the discussion on advance directives is not included in the health-care policy-making agenda. To encourage the discussion of advance directives, a study was designed to examine health professionals’ understanding and preferences related to advance directives. In addition, the study sought to explore the views of health care professionals of the application of Advance Directives (AD) in clinical practice in Lithuania.

**Methods:**

A cross-sectional survey was conducted by interviewing 478 health professionals based at major health care centers in Kaunas district, Lithuania. The design of the study included the use of a questionnaire developed for this study and validated by a pilot study. The collected data were analyzed using standard descriptive statistical methods.

**Results:**

The analysis of knowledge about AD revealed some statistically significant differences when comparing the respondents’ profession and gender. The analysis also indicated key emerging themes among respondents including tranquility of mind, the longest possible life expectancy and freedom of choice.

Further, the study findings revealed that more than half of the study participants preferred to express their will while alive by using advance directives.

**Conclusions:**

The study findings revealed a low level of knowledge on advance directives among health professionals. Most health professionals agreed that AD’s improved end-of-life decision making while the majority of physicians appreciated AD as the best tool for sharing responsibilities in clinical practice in Lithuania. More physicians than nurses preferred the presence of advance directives to support their decision making in end-of-life situations.

## Background

### The importance of advance directives

Rapidly ageing societies are one of the major challenges in the demographic history of humanity. It is scientifically estimated that the elderly population (aged over 65), will increase three fold between the years 1999 and 2050 [1]. The increase in the elderly population also presents an immediate challenge to health care systems.

Growing global life expectancy together with the prevalence of oncological and other terminal diseases has stimulated the discussion on end of life. End-of-life care refers to the total care of a person with advanced and incurable disease and aims to provide as good a quality of life as possible until death [2]. This increased the importance of the decisions taken in relation to health care.

On the other hand, the increasing capacity among patient’s to choose AD’s due to scientific advancements in health care reveals new ethical dilemmas. In particular, it renews the debate over changes in the role of the physician, nurse and other health professionals as well as increasing patients’ demands, especially in the decision-making at the end of life. A comparison of different countries shows that these considerations conflict more or less strongly depending on how autonomy is defined in a certain societies [3, 4]. Consequently, the concept of advance directives (AD) as one of the potential solutions to protect a patient’s rights, autonomy and dignity at the end of life have been widely discussed in recent decades. Generally, advance directives are defined as mechanisms by which individuals make known how they want decisions on medical treatment to be made when they can no longer make the decisions themselves [5]. So, advance directives inter alia can take the form of living wills, healthcare proxies, do-not-resuscitate orders, and enduring powers of attorney [6, 7].

However, advance directives are a complex issue and, more questions than answers have been raised so far regarding its legislation and application in clinical practice.

### Situation in Lithuania

Most Western countries demonstrate a patient-centered approach in their current health care systems. The aim of this is to protect a patient’s interests, respect his/her will, value preferences and priorities, as well as implementing their AD’s in emergency and end of life situations. An AD does not have legal standing in the Lithuania. However, these AD’s may be considered if available. Hospitals may wish promote this as a form of ‘patient-centered care’ with an aim to respect the wishes of patients at the end of their lives.

Advance directives are relatively new in Eastern and Central Europe. The Baltic States including Lithuania still lack scientific data related to the use of advance directives. AD represents a new term in Lithuania; it remains one of the EU members without regulation on AD. The patient’s right to refuse treatment and the issues of futile treatment are dealt only with by the Law on Patients’ Rights and Reimbursement for Harm in Lithuania [8].

Lithuania is therefore one of the few countries in the European Union (EU) where AD issues are still not on the of agenda of health-care policy-making. Previous studies indicated that (a) the public are not adequately informed on the use and application of AD in Lithuania; (b) the absence of a juridical basis regarding the application and legislation of AD causes various ethical or legal issues in end-of-life decision-making [9]. In particular, the issues of life-prolonging treatments (cardiopulmonary resuscitation (CPR), terminal condition, persistent vegetative state and do not resuscitate (DNR) or artificial nutrition and hydration require a revision and discussion regarding new legal instruments in medical decision-making. AD might be one of the means for solving potential clinical, ethical and legal problems [10].

The lack of information and initiatives from authorities has left the public unaware of the potential advantages and possibilities of AD. The concept and system of palliative care is also not well developed in Lithuania. Another concern is practices of “hidden euthanasia” within palliative care clinical settings. There is a question as to whether there is a need for AD’s especially in the care of the elderly and intensive care settings? Little is known on the expectations of those living in Lithuania regarding AD? A scientific debate regarding AD’s includes questions of its ethical outcomes and potential concerns on how the legislation of AD might affect real clinical practice. As a rule, such considerations emphasize the role of society, i.e. how AD is assessed and regarded by lay-people, patients and especially how AD is perceived by health professionals (physicians and nurses). End of life issues remain quite sensitive and are rarely approached by scientific researchers in Lithuania. Accordingly, the need for empirical studies in the early stages of the discussion of a national AD policy is one of the most important discussions in recent scientific literature [11, 12].

### AIM

To explore the understanding of health care professionals in Lithuania on Advance Directives and to explore the views of health care professionals of the application of Advance Directives in clinical practice in Lithuania.

## Methods

### Research design and sampling

A descriptive, cross-sectional, correlative design was applied in this study. The study was carried out in the Clinics and Oncologic Hospital of the Lithuanian University of Health Sciences (Kaunas, Lithuania) from 1 September –1 November in 2015. Overall, 478 health care professionals (out of 500 invited), participated and returned completed questionnaires (response rate – 95.8%.)

A convenience sampling design was employed. The sample included all nurses, physicians and pharmacists who were involved in the care and treatment of patients who require palliative care or were caring for patients with terminal illness.

### Instrument of the study

A descriptive, cross-sectional, correlative scholarly design was applied in this study. The study instrument was based on previous studies [9]. The questionnaire was tested in a pilot study. The questionnaire solicited personal and professional information and asked about views and attitudes about ADs. The sample for the pilot study consisted of 34 professional nurses and who have had daily contact with terminally ill patients in three major clinical settings. [9]. According to the results of the pilot study, some statements from the original study were adapted to suit the Lithuanian language version of the questionnaire.

The first part of the questionnaire included an assessment of preference on common themes representing fundamental ethical concepts such as autonomy, freedom of choice, beneficence and non-maleficence [13]. A Likert rating scale (from “absolutely important - 5” to “absolutely unimportant -1” was employed for the evaluation. For the statistical analysis, responses were generalized into three groups (first – “absolutely important” and “important”), second – “neutral”, third – “unimportant” and “absolutely unimportant”).

The second part of the questionnaire was designed to evaluate the attitudes of respondents towards the ethical issues and beliefs related to the application of advance directives. By using the Likert’s scale the data was again generalized into three groups in the statistical analysis, namely (“absolutely important and important”, “neutral”, and “unimportant” was merged with “absolutely unimportant”).

The final part of the instrument included basic socio-demographic characteristics as presented in Table 1.

**Table 1:** Social-demographic characteristics of respondents

Profession	Female		Male	
	%	N	%	N
Physician	16.9%	58	52.9%	72
Pharmacist	17.2%	59	33.8%	46
Nurse	65.9%	226	13.2%	18
Total	100.0	343	100.0	136
Clinical practice	%	N	%	N
Clinician	86.3%	296	76.5%	104
Not clinician	13.7%	47	23.5%	32
Total	100.0	343	100.0	136

### Demographic data

Age, gender and length of current employment, current work place and employment were provided by participants on the questionnaire.

### Data analysis

Study data were analyzed by using the SPSS for Windows 21.0 (Statistical Package for Social Sciences). The analysis of data included frequency of responses, and the statistical significance was estimated by p-value, when p > 0.05. Descriptive statistics included the calculation of the values of variables with 95% of confidence interval. A distribution of values emerged from the standard deviation. A statistical analysis of qualitative variables was conducted by using *χ* ^2^.

The assessment on the analysis of the reliability of the questionnaire was conducted using Cronbach’s alpha coefficient based on standardized values. This procedure was based on the Hedden (2004) scale; internal reliability assessment and recommendations showed that the scale is reliable if the Cronbach’s alpha value > 0.5 is used. The analysis indicated that the study complied with Cronbach’s alpha (- 0.863). The paper presents the respondents’ responses categorized into three groups for statistical purposes.

### Ethical considerations

The study was carried out in accordance with the ethical research principles of the Helsinki Declaration and the Code of Ethics approved by the Lithuanian Social Research Center (LSRC). Acquiring permission from the Lithuania Bioethics Committee was not necessary for this study.

Before the study was undertaken, hospital administration was informed and the ongoing study was approved and accordingly authorized. Informed consent was obtained from each survey respondent who agreed to participate.

Confidentiality was assured. Anonymity was maintained, as respondents were never asked for their names, surnames, or addresses. The collected data were summarized and reported in the aggregate and used only for scientific purposes.

## Results

### General knowledge about advance directives

The analysis of the knowledge of AD indicated a low level of knowledge about AD in general among the study participants. The study revealed only 16.7% of the respondents confirmed their knowledge of what was meant by using the “Advance directives”, while more than half of the respondents stated their limited knowledge level about AD.

The analysis of the knowledge about AD also disclosed some statistically significant differences comparing the respondents’ profession and gender (Table 2). The physicians were more familiar with the content of AD than nurses and pharmacists.

**Table 2:** The respondents’ knowledge of advance directives (AD)

Statements	Profession				
	Physicians	Pharmacists	Nurses	Common level of knowledge	
	Absolutely important/important				
	%	%	%	%	
I clearly understood what is AD	24.6	12.4^a^	13.9^a^	16.7	*χ* ^2^ = 23.84 df = 4 *p* = 0.000
I have limited understanding about AD	57.7	57.1	45.9	51.7	
I don’t know what is AD	57.7	30.5^b^	40.2^b^	31.4	

^a^Statistically significant comparing pharmacists to physicians

^b^Statistically significant comparing nurses to physicians

### Value orientation among health professionals

The general evaluation of respondents’ value orientation in health care was conducted by calculating mean scores based on a scale from 1 (“absolutely unimportant”) to 5 (“absolutely important”). The analysis revealed major themes emerging including tranquility of mind (2.9), the longest possible life expectancy and the freedom of choice (both 2.54), then the absence of suffering (2.52), and the best possible quality of life (2.56).

By analyzing the importance of values depending on profession and gender, we revealed that the most important values in clinical activities such as a longer life expectancy and others were more frequently identified by female physicians and differed significantly when comparing them to nursing females (see Table 3). Moreover, female pharmacists compared to female physicians and nurses were more likely to report that one of the most important themes as tranquility of mind. In the male group of respondents, the statistics showed no significant differences at all.

**Table 3:** Importance of value-orientation according to respondents ‘profession and gender

Values		Physician	Pharmacist	Nurse	
		Absolutely important/importance			
		%	%	%	
Longest possible life expectancy	Female	87.9^a^	84.7	33.2	*χ* ^2^ = 89.4 df = 4 *p* = 0.000
	Male	87.5	80.4	77.8	*χ* ^2^ = 2.4 df = 4 *p* = 0.645
Tranquility of mind	Female	60.3	69.5^a^	25.2	*χ* ^2^ = 86.1 df = 4 *p* = 0.000
	Male	52.8	54.3	72.2	*χ* ^2^ = 2.4 df = 4 *p* = 0.661
Absence of suffering	Female	93.0^a^	86.4	33.2	*χ* ^2^ = 117.1 df = 4 *p* = 0.000
	Male	94.4	91.3	88.9	*χ* ^2^ = 6.09 df = 4 *p* = 0.192
Ability of free choice	Female	96.6^a^	91.5	31.4	*χ* ^2^ = 129.6 df = 4 *p* = 0.000
	Male	86.1	93.5	88.9	*χ* ^2^ = 2.07 df = 4 *p* = 0.723
Best possible quality of life	Female	87.9^a^	91.5	29.6	*χ* ^2^ = 121.3 df = 4 *p* = 0.000
	Male	95.8	91.3	83.3	*χ* ^2^ = 5.61 df = 4 *p* = 0.230

^a^Statistically significant comparing physicians and nurses, and comparing pharmacists and nurses

### The perceptions and beliefs regarding advance directives

The study also revealed that more than half of the study participants preferred to express their wishes prior to the onset of illness by using an advance directive. Ethical and legally legitimated AD was recognized by more than half of our HCPs (see Table 4). Statistically significant results also determined that female physicians more frequently agreed with the statement that the purpose to discuss end of life issues during critical illness is ethical for patients, compared to the opinion of female nurses (respectively 81.1% and 56.7%). Moreover, the statement that AD is the right way to deal with the potential problems of passive euthanasia was more agreed on by female physicians (70.7%) than by female nurses (59.7%). The vast majority of health care professionals (both female and male) agreed with the provision that the AD would help harmonize the sharing of responsibility between the health professional and the patient. Female nurses compared to female physicians significantly less often appreciate that the application of advance directives might be helpful for health professionals to manage the end of life decision making. And finally, more than half of the respondents, both female and male, would prefer to see the AD as legally binding in the health care system of the Lithuania (see Table 4).

**Table 4:** Perceptions and beliefs to advance directives according to respondents’profession and gender

Statement	Gender	Physician	Pharmacist	Nurse	
		Positive perceptions (absolutely important and important)			
		%	%	%	
I prefer to express my will by using legitimated advance directives	Female	74.1	69.5	59.7	*χ* ^2^ = 10.01 df = 3 *p* = 0.124
	Male	69.4	71.8	72.2	*χ* ^2^ = 0,96 df = 6 *p* = 0.987
Proposal to consider on the patient’s end of life issues in emergency clinical cases is ethically justified	Female	81.1^a^	71.2	56.7	*χ* ^2^ = 22.0 df = 6 *p* = 0.001
	Male	73.6	80.4	38.9	*χ* ^2^ = 22.0 df = 6 *p* = 0.001
Advance directives might be helpful to balance the sharing responsibility between health professional and patient	Female	63.8	55.9	49.6	*χ* ^2^ = 8.02 df = 6 *p* = 0.236
	Male	66.6	67.3	61.1	*χ* ^2^ = 6.53 df = 6 *p* = 0.366
Advance directives might be an appropriate mean to resolve the issues of passive euthanasia and assisted suicide	Female	70.7	62.6	59.7	*χ*2 = 11.7 df = 6 *p* = 0.068
	Male	65.3	63.0	72.2	*χ*2 = 1.44 df = 6 *p* = 0.963
Application of advance directives might be helpful to health professionals in managing end of life decisions	Female	79.3	88.1	66.8^b^	*χ* ^2^ = 18.45 df = 8 *p* = 0.018
	Male	77.7	80.5	77.7	*χ* ^2^ = 1.48 df = 6 *p* = 0.960
Advance directives should be legally enforced in medical practice	Female	70.7	71.2	64.6	*χ* ^2^ = 3.6 df = 6 *p* = 0.725
	Female	58.3	54.3	66.7	*χ* ^2^ = 2.22 df = 6 *p* = 0.897

^a^Statistically significant comparing physicians and nurses

^b^Statistically significant comparing nurses and physicians

## Discussion

### Knowledge about AD

Advances directives are used to express the personal will at the end of life. On other hand, family members of the patient as well as health professionals (including physicians and nurses), play an important role in the planning of a patient’s end of life decisions [12]. The patients are to debate the end of life options quite frequently with those who surround them; hence AD might be regarded as an appropriate tool to protect their autonomy and dignity ethically and legally in this kind of debate [14]. However, to apply such opportunities, knowledge about AD is required. Our study revealed that only 16.7% of the respondents were familiar with the AD concept, more than half of all respondents just heard about it, but were not familiar with it. Statistically high level of knowledge was determined among physicians compared with nurses and pharmacists, but the level of knowledge related to AD is still low among Lithuanian health professionals.

The findings of our study were quite similar to the findings in other similar studies, for example, the level of knowledge about AD among nursing personnel [15]. In particular, the Duke and Thompson study showed a lack of common and legal knowledge about AD and similarly, poor knowledge and little experience on AD was reported among nurses in New Zealand [16].

In contrast to these findings, the analysis of other studies indicated more positive results. For instance, the study by Ryan et al (2012) reported more than nearly two-thirds of nurses being knowledgeable about AD [17], while the study by Jezewski et al. reported 70% of nurses to have general awareness of AD and 53% of them had legal knowledge on AD [18, 19].

Accordingly, the different results of the level of AD awareness might be associated to the legal status of AD as well as to the social, economical or even cultural factors of the country.

#### Value orientation

The discussion on the application of AD to clinical practice cannot be separated from the ethics and value orientation of health professionals, especially when faced with the emergent end-of-life situations. Fundamental values of medicine can be summarized by basic ethical principles such as respect for the person, beneficence, non-maleficence, and justice; therefore, their application by respecting patient autonomy or responsibility for appropriate decisions about treatment should be upheld in practice [13]. In the case of our study, health professionals preferred the absence of pain and suffering (presumably, the application of the principle of beneficence) followed by freedom of choice (patient autonomy) and better life expectancy (non-maleficence).

### Attitudes and beliefs

Attitudes, beliefs and values of physicians, nurses and other health care providers contribute significantly to the options in honoring a patient’s wishes for end-of-life care [20, 21]. In this context, our study revealed that the major part of respondents (both male and female) would rather express their living will on the end of life by using AD. Presumably, they would also respect the patient’s will, expressed through AD if it were legally binding.

Another important question – Is the suggestion to negotiate the end of life issues with patients ethical from a professional point of view? The study from Canadian scientists disclosed that only 19% of physicians would discuss the AD issues in general, while more than half of them reported they would not to comply with their patients’ (AD) will expressed in in certain situation [22]. Similar results were reported by Japanese scholars: 55% of study participants (physicians) support AD application, while 33% of them had a possibility to discuss it with their patients in clinical practice [23].

Moreover, a number of studies about nurses’ experiences revealed that discussion about the end of life is regarded as a cultural taboo, hence many patients were not eager to talk about it [24–26]. Additionally, an Australian study constituted a more positive approach to AD and its application in emergency cases, and AD was regarded as the appropriate instrument to reduce tension and manage potential conflicts among patients, family members and health professionals [27].

The findings of our study correlate with the reports from other countries. The majority of respondents believed that the discussion over end-of-life decisions together with patients could be regarded as ethical sound and acceptable. This leads us to interpret health professionals as tending to respect a patient’s autonomy and to support death with dignity, even though the concept of autonomy is relatively new in Lithuania.

Another question - who should be more responsible for the end-of-life decisions – was presented to the respondents of our study. This was a complex and very sensitive issue for health professionals, and some studies confirmed it as a stressful debate depending on individual values, work experience, the value orientation and communication competencies [21].

Numerous studies disclosed quite varying attitudes in nurses’ on sharing responsibilities over AD application in practice. Some of the nursing personnel claimed such a responsibility is not a part of their competence and consequently they would prefer not to take it upon themselves to discuss this with their patients [27]. Meanwhile, some studies claim AD should be part of nurses’ practice, but it should not be a priority when caring for patients [28]. In other studies, nurses viewed the AD application as a task for a multidisciplinary team, but the sharing of responsibility was not clear so far [29–31]. Accordingly, more than half of the respondents in our study indicated the management of AD as the sharing of moral responsibility between health professional and the patient. In conclusion, sharing responsibilities over ethically sensitive issues remained to be open for further scientific debate and discussion.

More questions relating to AD and its application remain to be clarified. For instance, are AD’s helpful in health care practice in general or would it complicate routine practices of health professionals? For instance, most nurses in New Zealand agreed with the idea that AD might enhance a more effective care at the end of life care service [31]. Another study conducted in the European Amyotroph Lateral Scler centers revealed, 78% positive beliefs about AD effectiveness in the end-of-life care and 55% of them reported AD’s being discussed in practice [32].

Overall, our study reported similar findings which reflect the tendencies related in other previously mentioned scientific discussions over AD. The majority of our respondents stated their positive expectations related to AD perspectives while making the end-of-life decisions in future clinical practice.

## Conclusions

The analysis of the study results revealed the low level of knowledge on advance directives among health professionals. The absence of suffering, freedom of choice and longest possible life expectancy were the dominant discourse among all health professionals. Most of the health professionals agreed on the positive AD influence while improving the end-of-life decision making; most physicians appreciated AD as the best tool for sharing the responsibilities in clinical practice in Lithuania.

We also observed that physicians were significantly more positive compared to nurses in assessing the potential benefits by application of advance directives to manage the end-of-life decision making.

### Strength and limitations

The study included the participation of physicians, nurses and pharmacists, thereby addressing an interdisciplinary problem which contributes to the strength of the study. Additionally, it was one of the first such studies which aimed at revealing the attitudes of health professionals towards advance directives and its implications in clinical practice in Lithuania.

Meanwhile, a limitation might be that the study presents merely the attitudes and preferences reported by health professionals, but not by patients’ or citizens of the country.

Another limitation of our study is the reliability of self – reports while assessing knowledge of respondents about AD. There is scope for further and differently designed studies (e.g. qualitative study) to explore this emerging issue on AD’s observed in health care.

## Abbreviations

AD: Advance directives
EoL: End of life
EU: European Union
HCPs: Health care professionals

## References

[1] Health data and statics. Available: http://www.who.int/healthinfo/statistics/en/.

[2] Department of Health. End of Life Care Strategy: Quality Markers Consultation. 2008. Available: https://www.gov.uk/government/organisations/department-of-health.

[3] Horn JR (2014). Advance Directives in English and French Law: Different Concepts, Different Values, Different Societies. Health Care Anal.

[4] Beširevic V (2010). End-of-life care in the 21st century: advance directives in universal rights. Bioethics.

[5] Evans N, Bausewein C, Menaca A, Erin A, Higginson IJ, Harding R, Pool R, Gysels M (2012). A critical review of advance directives in Germany: Attitudes, use and healthcare professionals’ compliance. Patient Educ Couns.

[6] Lorda PS, Velázquez TMI, Cantalejo BIM (2008). Advance Directives in Spain: Perspectives from a Medical Bioethicist Approach. Bioethics.

[7] The World Medical Association Statement On Advance Directives (“Living Wills”) by the WMA General Assembly, Helsinki 2003. https://www.wma.net/policy/.

[8] Lietuvos Respublikos Pacientų teisių ir žalos sveikatai atlyginimo įstatymas. 1996 m. spalio 3 d. Nr. I-1562. Nauja istatymo redakcija nuo 2010 m. kovo. Nr.XI – 499

[9] Blazeviciene A, Peicius E (2011). Nurses’ Attitudes Toward Advance Directives in Lithuania. NERP (Kaunas).

[10] Johns JL (1996). Advance Directives and Opportunities for Nurses. Image J Nurs Sch.

[11] Solomon MZ, O’Donnell L, Jennings B, Guilfoy V, Wolf SM, Nolan K, Jackson R, Koch-Weser D, Donnelley S (1993). Decisions near the End of Life: Professional views on Life Sustaining Treatments. Am J Public Health.

[12] Cogo SB, Lerch VL (2015). Anticipated directives and living will for terminal patients: an integrative review. Rev Bras Enferm.

[13] Beauchamp TL, Childress JF (2001). Principles of biomedical ethics.

[14] Flores TR, Mato SA, Rivero AP, Galan ATM (2013). Knowledge and attitudes about advance directives on physicians and nurses. Aten Primaria.

[15] Oliver D, Campbell C, Sloan R (2007). End-of-life care and decision making in ALS/MND: A cross-cultural study. Amyotroph Lateral Scler.

[16] Duke G, Thompson S (2007). Knowledge, attitudes and practices of nursing personnel regarding advance directives. Int J Palliat Nurs.

[17] Frey R, Raphael D, Bellamy G, Gott M (2014). Advance care planning for Maori, Pacific and Asian people: the views of New Zealand healthcare professionals. Health Soc Care Community.

[18] Ryan D, Jezewski AM (2012). Knowledge, Attitudes, Experiences, and Confidence of Nurses in Completing Advance Directives: A Systematic Synthesis of Three Studies. J Nurs Res.

[19] Jezewski AM, Brown JK, Wu YB, Meeker MA, Feng J, Bu X (2005). Oncology nurses’ knowledge, attitudes, and experiences regarding advance directives. Oncol Nurs Forum.

[20] Teno MT, Stevens M, Spernak S, Lynn J (1998). Role of Written Advance Directives in Decision Making. J Gen Intern Med.

[21] Dobratz MC (2005). Gently into the light: a call for the critical analysis of end-of-life outcomes. ANS Adv Nurs Sci.

[22] Hughes DL, Singer PA (1992). Family physicians’ attitudes toward advance directives. CMAJ.

[23] Ke LS, Huang X, O’Connor M, Lee S (2015). Nurses’ views regarding implementing advance care planning for older people: a systematic review and synthesis of qualitative studies. J Clin Nurs.

[24] Boot M, Wilson C. Clinical nurse specialists’ perspectives on advance care planning conversations: a qualitative study. Int J Palliat Nurs. 2014;20(1):9-14. 10.12968/ijpn.2014.20.1.9 24464168

[25] Shinmi K, Yunjung L. Korean nurses’ attitudes to good and bad death, life-sustaining treatment and advance directives. Nurs Ethics. 2003;10(6):625-35. 10.1191/0969733003ne652oa 14650481

[26] Lipson AR, Hausman AJ, Higgins PA, Burant CJ (2004). Knowledge, attitudes, and predictors of advance directive discussions of registered nurses. West J Nurs Res.

[27] Johnson C, Singer R, Masso M, Sellars M, Silvester W (2015). Palliative care health professionals’ experiences of caring for patients with advance care directives. Aust Health Rev.

[28] Seymour J, Almack K, Kennedy S (2010). Implementing advance care planning: a qualitative study of community nurses’ views and experiences. BioMed Central Palliat Care.

[29] Baughman KR, Aultman J, Hazelett S, Palmisano B, O’Neill A, Ludwick R, Sanders M (2012). Managing in the trenches of consumer care: the challenges of understanding and initiating the advance care planning process. J Gerontol Soc Work.

[30] Jezewski MA, Meeker MA, Schrader M (2003). Voices of oncology nurses: what is needed to assist patients with advance directives. Cancer Nurs.

[31] Robinson L, Dickinson C, Bamford C, Clark A, Hughes J, Exley C (2013). A qualitative study: professionals’ experiences of advance care planning in dementia and palliative care, ‘a good idea in theory but. Palliat Med.

[32] Malpas JP (2011). Advance directives and older people: ethical challenges in the promotion of advance directives in New Zealand. J Med Ethics.

